# MicroRNA regulation of Transthyretin in trophoblast differentiation and Intra-Uterine Growth Restriction

**DOI:** 10.1038/s41598-017-16566-0

**Published:** 2017-11-29

**Authors:** Sarbani Saha, Shreeta Chakraborty, Agnihotri Bhattacharya, Arati Biswas, Rupasri Ain

**Affiliations:** 1 Division of Cell Biology and Physiology, CSIR-Indian Institute of Chemical Biology, Kolkata, 700032 India; 2 Calcutta National Medical College, Kolkata, 700014 India

## Abstract

Placental trophoblast cells produce various cytokines, transporters vital to normal embryogenesis. Transthyretin (TTR) aids trans-placental passage of maternal thyroxin (TH) to fetal circulation. Inadequate TH delivery leads to developmental abnormality. Regulation of TTR biosynthesis in placenta is critical for normal embryo development. We showed here that TTR transcripts were expressed more in fetal placenta. Using bioinformatic analysis and confirmation with dual-luciferase reporter assays, we found that miR-200a-3p and miR-141-3p inhibited TTR expression by directly binding to the 3′UTR of TTR, which is reversed by mutation in the microRNA binding site. Differentiation of human trophoblast BeWo cells was associated with decreased TTR transcript and protein levels with concomitant increase in the levels of both microRNAs. Interestingly, ectopic overexpression of the microRNA mimics abrogated thyroxin uptake by BeWo cells, which was reversed by the corresponding inhibitors. Furthermore, in a rat model of intra-uterine growth restriction (IUGR), TTR expression decreased significantly in placenta with reciprocal rise in miR-141-3p but not 200a-3p. In human IUGR placenta, TTR transcript and protein levels were significantly lower associated with high expression of miR-141-3p but not 200a-3p. These data provides new insight into physiological role of miR-141-3p in regulating TTR during trophoblast differentiation and IUGR.

## Introduction

Placenta is a highly specialized extra-embryonic tissue which plays essential roles during the course of pregnancy, including fetal development, nourishment, protection, gas exchange and many others. Trophoblast cells, the parenchymal cells of the placenta, produce hormones and cytokines that regulate the activities of the maternal environment and they possess transport machinery that facilitates the delivery of hormones and nutrients to the fetus. One such important transporter is transthyretin (TTR) that aids trans-placental passage of maternal thyroxin (T_4_) to fetal circulation. The fetal thyroid acquires the capability of  Thyroid hormone (TH) secretion from twelfth gestation week. So, till second trimester the fetus relies entirely on transplacental delivery of maternal THs^1,2^. Even after the beginning of fetal TH synthesis, transfer of the maternal TH to the fetal vasculature still continues throughout the pregnancy, especially in case of hypothyroidism^3,4^. These thyroid hormones are one of the critical factors for proper fetal development, especially, that of the central nervous system of the fetus. Slight insufficiency of maternal thyroid hormone supply would have detrimental effects on fetal neural health. Even mild variations in TH levels during pregnancy may cause reduced intelligence quotient in children^5–7^. These findings portray the importance of sufficient delivery of maternal thyroid hormones to the fetus during pregnancy.

Several thyroid hormone binding proteins are synthesized in human placenta. Among these proteins, thyroxin binding globulin (TBG), transthyretin (TTR) and albumin (ALB) have been shown to bind with thyroxin (T_4_) with high affinities^8^. TTR is responsible for the transport of 15% of circulating T_4_^9^, but in contrast, TTR is the primary carrier of T_4_ in the cerebrospinal fluid as it is the only thyroid hormone binding plasma protein synthesized in the brain^10^. Transthyretin, first discovered in 1942, in human serum and cerebrospinal fluid, was previously known as prealbumin as it migrated faster than serum albumin during electrophoresis of whole plasma^11,12^. The protein was named as transthyretin in the year 1981, because of its role in the transport of thyroxin hormone (T_4_) and retinol. TTR is primarily synthesized in the liver, eye and choroid plexus of brain but it has also been found to be synthesized from placental villous trophoblast^13,14^. During pregnancy, TTR is expressed from placental tissue from 6 weeks of gestation and increased gradually in a time dependent manner throughout early pregnancy (6–13 weeks). Expression of TTR reaches its peak around the beginning of second trimester of pregnancy and remains at that level till term^15^. TTR is secreted from placental villous trophoblast at the maternal-placental interface^8^. TTR tetramers bind to the thyroxin hormone (T_4_) present in the maternal blood and this binding stabilizes the TTR tetramer. TTR-T_4_ conjugate is endocytosed by placental trophoblast cells^16^. The shuttling of maternal thyroxin to fetal vasculature is regulated by several critical factors like concentration of TTR and T_4_, oxygen level etc^16,17^.

Besides its important roles in development, TTR is also associated to the pathophysiology of several diseases. In early pregnancy loss in human, TTR protein is found to be expressed at a lower level from placental villous trophoblast^18^. In patients with preeclampsia (PE), a pregnancy disorder characterized by high blood pressure and proteinuria, dissociation of TTR tetramer leads to formation of partially unfolded monomers which aggregate to form amyloid fibrils. Deposition of those fibrils in the placental tissue as well as maternal vasculature facilitates the establishment of the disease^19^. Another groups also found that maternal serum of patients suffering from severe PE has a lower level of TTR which may affect the normal development of the growing fetus^20–22^. In a very recent study, expression of TTR was found to be down regulated in placental tissues from severe PE patients compared to control ones^23^. These findings strongly suggest the fact that PE is associated with reduced levels of TTR. In contrast, it was reported that TTR expression of placental tissue was higher in patients with PE and intrauterine growth retardation (IUGR) when compared to normal placentas^24,25^. Both these studies were based on low sample size, which may be a cause of this discrepancy. It is therefore evident that biogenesis and maintenance of TTR levels during pregnancy is essential for normal fetal development.

MicroRNAs constitute a group of small (21–25 nt) endogenous non-coding single stranded RNAs which regulate gene expression post-transcriptionally by complimentary pairing of 6–8 nucleotide (seed sequence) with the target mRNAs. Binding of microRNAs to respective mRNA leads to formation of RISC complex resulting in translational repression or degradation of target mRNAs^26,27^. In mammals, microRNAs are highly conserved across species^28^. Different studies have shown that one third of human genes are controlled by different microRNAs^29^, thereby suggesting the potential role of microRNAs in many physiological processes. In recent years, microRNAs have been shown to participate in various fundamental cellular processes including cell development, proliferation, metabolism, apoptosis, hormone signaling, stem cell maintenance and differentiation^26,30–34^. It has been reported that microRNAs are also involved in a variety of human diseases including various pregnancy related disorders^35–37^. MicroRNA microarray and quantitative PCR based expression analyses have revealed that many unique microRNAs are expressed in human placenta^38–40^. Analyses of microRNA expression in preeclamptic versus normal placentas have suggested the role of microRNAs in regulation of various genes during the pathogenesis of preeclampsia^41^. But the regulating effect of microRNAs on IUGR related genes is poorly understood. Recent reports have shown that, miR-141 is secreted from placental trophoblast and is up regulated in the third trimester (29–40 weeks) of pregnancy, but its expression is increased abnormally in preeclamptic placentas when compared with normal placentas^42,43^. Upregulation of this microRNA was also found in placental tissue from FGR patients where miR-141 was found to down regulate E2F3 and PLAG1^44^.

Although TTR, expressed by the placental trophoblast cells, plays very important roles in fetal development but so far no microRNA has been reported to regulate TTR biogenesis during trophoblast differentiation. Understanding of the role of microRNAs in post-transcriptional regulation of TTR gene expression in the context of trophoblast cell development will provide novel insights into pathophysiological conditions with compromised fetal development. We, therefore, sought to investigate the role of conserved microRNAs in regulating TTR gene in trophoblast differentiation and in IUGR. Using various bioinformatic software, binding sites of two microRNAs, miR-141-3p and miR-200a-3p were identified to be present in the 3′-UTR of human TTR and were found to be conserved across species. Therefore, the role of these two microRNAs was investigated in regulating human TTR during trophoblast development and IUGR.

## Results

### TTR mRNA is expressed in developing rat placenta and its cellular source is not restricted to any particular trophoblast lineage

**Figure 1:**
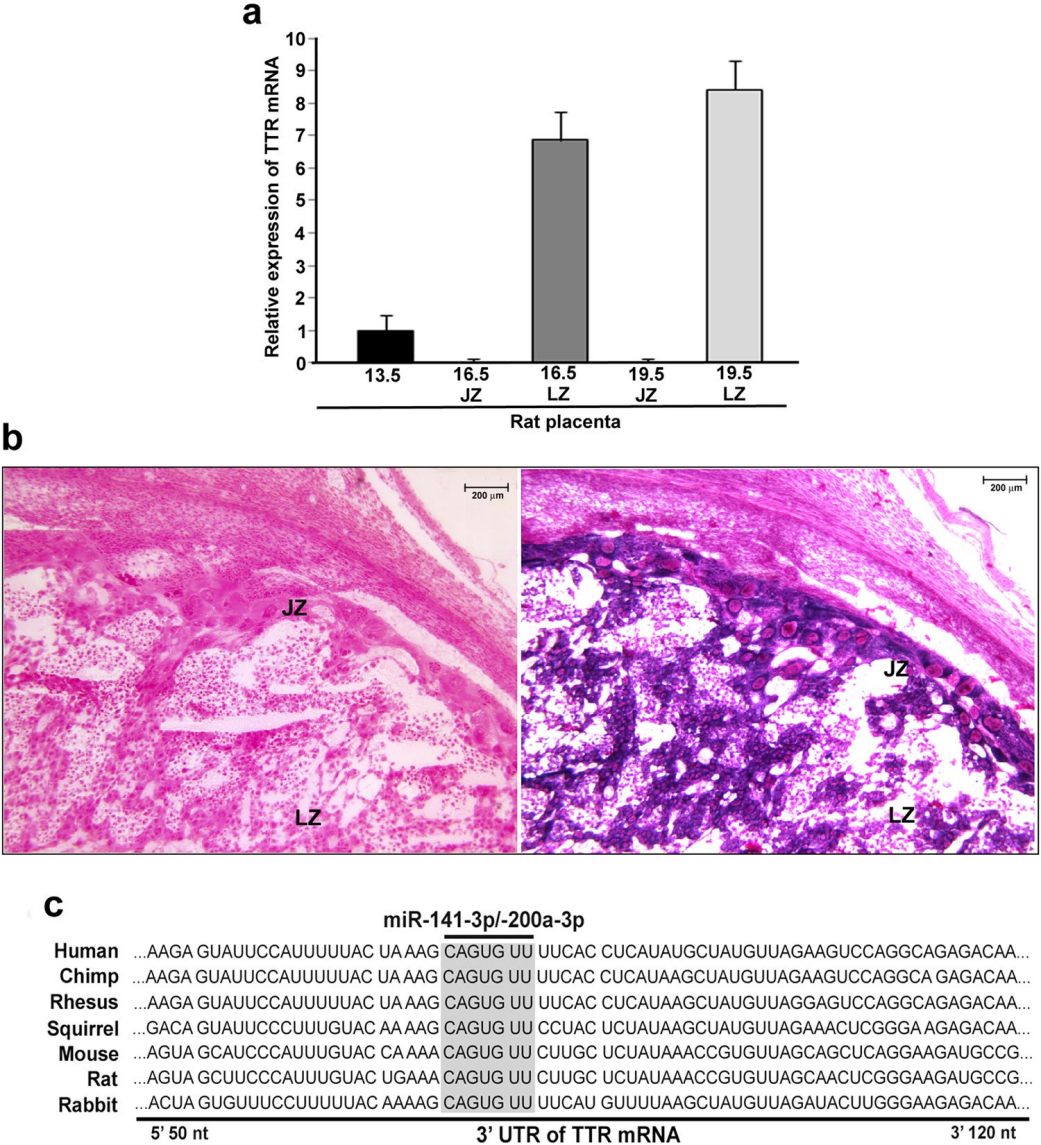
Cellular source of TTR in developing rat placenta and miR-200a-3p, miR-141-3p binding sites on 3′-UTR of TTR mRNA (**a**) Quantitative real time PCR analysis of TTR mRNA from rat placental samples at different days of gestation. JZ and LZ: Junctional zone and Labyrinth zone of the placenta respectively. (**b**) *In situ* localization of TTR mRNA in rat placenta on gestation day 16.5. Rat placental tissues were isolated on gestation day 16.5. 10-micron cryo-sections of placental tissues were prepared and hybridized to digoxigenin-labeled sense (left panel) and anti-sense (right panel) riboprobes for TTR. Image was taken at 100X magnification. Purple color indicates TTR mRNA in cytosol and nuclear fast red staining (pink color) indicates counter staining of the tissue sections. JZ and LZ: Junctional zone and Labyrinth zone of the placenta respectively. (**c**) Sequence of 3′-UTR of TTR transcript showing binding site of miR-200a-3p and miR-141-3p is conserved across species.

Temporal expression and relative abundance of TTR transcript in rat placenta was assessed by real time PCR analyses (Fig. 1a). Expression of TTR transcript was found in rat placental tissue at day 13.5, when junctional zone and labyrinth zone cannot be surgically separated (Ct value is 20.7). At later stages of gestation, TTR mRNA was expressed more in labyrinth zone (Ct values in D16.5 and D19.5 are 18.2 and 18.3, respectively), as compared to the junctional zone of the placenta (Ct values in D16.5 and D19.5 are 26.8 and 26.5, respectively). Since placental tissue contains various trophoblast lineages, the cellular site of TTR synthesis was determined by *in situ* hybridization analyses using sense and antisense probes on D16.5 utero-placental tissue sections (Fig. 1b). Sense probes did not show detectable hybridization in any cell type of the placenta. TTR mRNA was localized in labyrinthine trophoblast as well as the giant cells and spongiotrophoblast cells of the placenta. However, the intensity of spongiotrophoblast staining was negligible compared to giant cells and labyrinthine trophoblast cells.

### Regulatory microRNAs targeting 3′-UTR of TTR

Successful use of RNA interference to down-regulate cortical transthyretin prompted us to look for endogenous microRNAs regulating TTR expression vital to normal embryogenesis. Bioinformatic analysis was performed to identify the microRNA(s) that bind to 3′-UTR of TTR and thus potentially lead to post-transcriptional regulation of TTR during development and disease. Output from various microRNA prediction tools has been revealed that two members of miR-200 family, miR-200a-3p and miR-141-3p have conserved binding sites on the 3′UTR of TTR mRNA. Both miR-141-3p and miR-200a-3p share a common microRNA response element (MRE) on the 3′UTR of TTR transcript, which is highly conserved across species including humans (Fig. 1c). Interestingly, expression of these microRNAs in developing rat placental samples, as assessed by TaqMan assay was very low (Ct values ≥29.5 using U6 snRNA as internal control). This reciprocal expression pattern of TTR and the microRNAs indicate that these microRNAs might regulate TTR expression *in vivo*.

**Figure 2:**
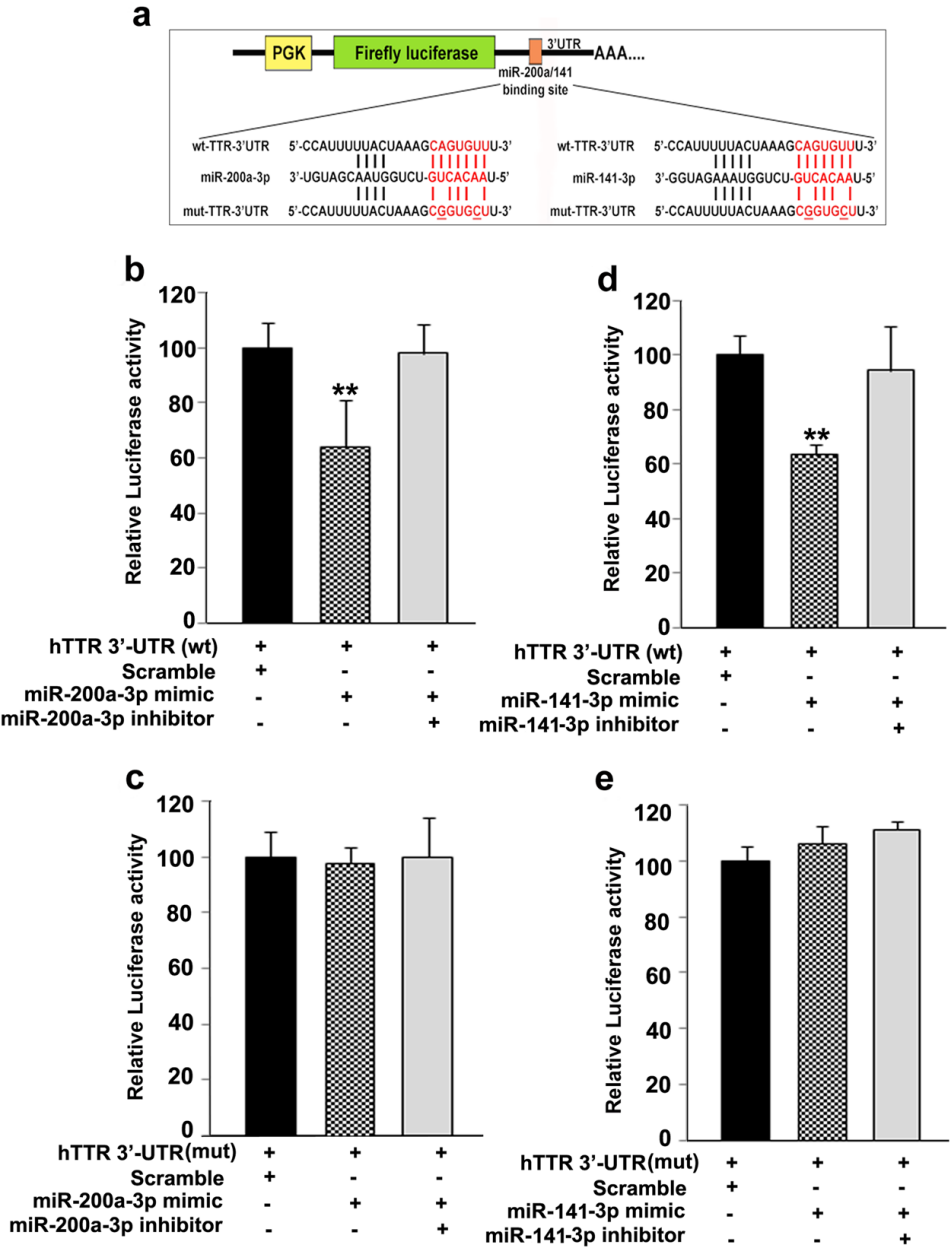
MiR-200a-3p and miR-141-3p directly target TTR 3′-UTR. (**a**) Schematic diagram of pmirGLO-hTTR-3′UTR reporter construct with wild-type (wt) and mutated (mut) binding site for miR-200a-3p and miR-141-3p. Mutated version of hTTR 3′UTR (5′-CGGTGCT-3′) were generated by introducing two point mutations in the seed region (5′-CAGTGTT-3′) of miR-141-3p and miR-200a-3p. (**b**–**d**) Wild type (wt) or mutated (mut) pmir-GLO-TTR 3′-UTR plasmid was co-transfected to HEK-293 cells with microRNA mimic alone or in a combination of mimic and respective inhibitor. Scrambled mimic microRNA was used as control. Levels of firefly and renilla luciferase activities were determined 24 hours post transfection. Relative luiciferase activity was determined using Renilla luciferase activity for transfection normalization. Data are presented as relative luciferase activity (mean ± SEM) from three different experiments (n = 3). (**b**) Repressive effect of miR-200a-3p mimic on ‘wild type’ TTR-3′UTR and its reversal by the specific inhibitor. (**c**) No effect of the miR-200a-3p mimic on TTR-3′UTR containing ‘mutated’ binding site for miR-200a-3p. (**d**,**e**) Similar results obtained with miR-141-3p. ANOVA followed by Newman-Keuls multiple comparison test were used to calculate the significance, where asterisks (**) indicate significant differences (p < 0.005) compared with scramble transfected control cells.

To validate whether TTR mRNA is a direct target of miR-200a-3p and miR-141-3p, dual luciferase reporter assay was performed in HEK293 cells. Two luciferase reporter constructs were made for this purpose. In the first construct 14–263 nucleotide of 3′UTR of TTR containing the putative MRE for miR-200a-3p and miR-141-3p (5′-CAGTGTT-3′) was cloned downstream of the firefly luciferease reporter in the pmirGLO plasmid and was denoted as ‘wild type’. The second one is the same fragment of ‘wild type’ TTR 3′UTR, except having two point mutations in the MRE for these two microRNAs (5′-CGGTGCT-3′) and denoted as ‘mutated’ (Fig. 2a). Co-transfection of the reporter containing wild type 3′-UTR of TTR along with either miR200a-3p or miR141-3p mimic decreased the relative luciferase activity by almost 40%, as compared to scramble microRNA mimic control (Fig. 2b and d). Successful reversal of this suppression phenomenon was observed with the addition of inhibitors for miR-200a-3p and miR-141-3p along with the respective mimics (Fig. 2b and d). As expected, co-transfection of the reporter containing mutated 3′-UTR of TTR along with either miR200a-3p or miR141-3p mimic did not have any effect on relative luciferase activity as compared to scramble control (Fig. 2c and e).

### Reciprocal expression of TTR and the regulatory microRNAs during human trophoblast cell differentiation

**Figure 3:**
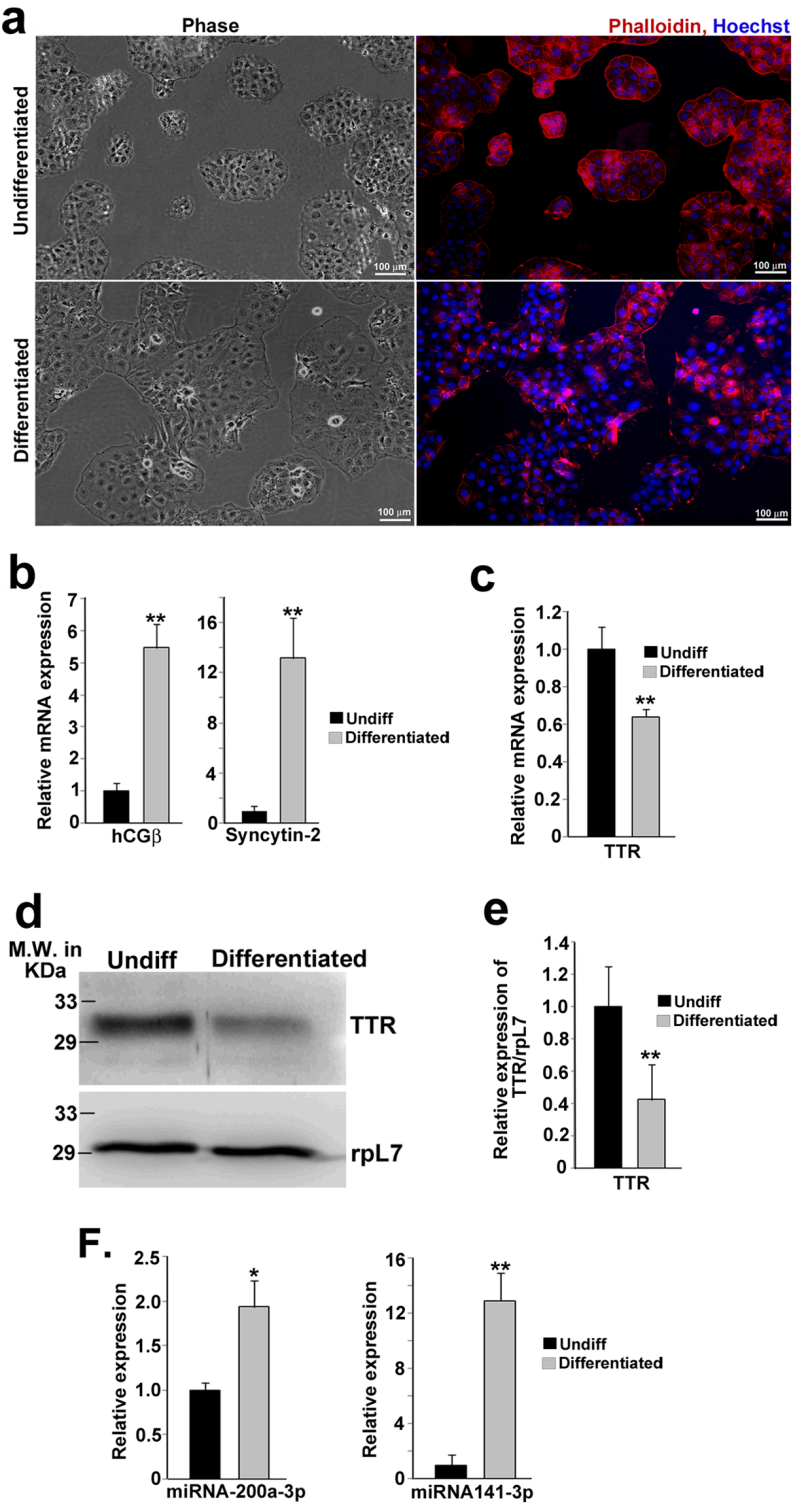
Differentiation of human trophoblast cells (BeWo) is associated with reciprocal expression of hTTR and miR-200a-3p, miR-141-3p. BeWo cells were treated with 250 µM of 8-Bromo-cAMP for 72 hours to induce differentiation (syncytialization). (**a**) Photomicrograph of undifferentiated and differentiated BeWo cells showing syncytialization upon 8-Bromo-cAMP treatment. Left panel: phase contrast, Right panel: Actin filaments labeled with DyLight™ 554 Phalloidin (red) and the nucleus stained with Hoechst dye (blue). (**b**) Quantitative real time PCR for differentiation markers, hCG-β and hSyncytin-2 in control and 8-Bromo-cAMP treated BeWo cells. (**c**) Quantitative real time PCR analysis for hTTR transcript in control and 8-Bromo-cAMP treated BeWo cells. (**d**) Western blot analysis of hTTR protein expression in control and 8-Bromo-cAMP treated BeWo cells. (**e**) Quantification by ImageJ software of the proteins relative to rpL7 using three biological replicates from d. (**f**) TaqMan assays for miR-200a-3p and miR-141-3p in control and 8-Bromo-cAMP treated BeWo cells. *p < 0.05, **p < 0.005.

Fusion of cytotrophoblast cells to form syncytial trophoblast cells is a hallmark event in human placental development and maturation. In human trophoblast cell line, BeWo, induction of differentiation leads to formation of syncytial trophoblast cells. These trophoblast cells can be used as an *ex vivo* model system for studying the effects of miR-141-3p and miR-200a-3p on endogenous TTR during trophoblast differentiation. BeWo cells were cultured and allowed to differentiate by treating the cells with 8-Bromo-cAMP for 72 h. Formation of multinucleated syncytium was visualized by staining the cytoskeletal protein F-actin with Dylight-conjugated phalloidin (Fig. 3a). Massive up-regulation of hCG-β and Syncytin-2 transcripts, which are abundantly expressed in syncytiotrophoblast cells, as assessed by real time PCR further confirmed differentiation of BeWo cells to syncytiotrophoblast cells (Fig. 3b).

Following characterization of the differentiated trophoblast cells, TTR expression pattern was analyzed in undifferentiated and differentiated trophoblast cells by real-time PCR and western blot analyses. Both TTR transcript and protein decreased significantly in differentiated trophoblast cells (Fig. 3c–e). In line with our previous results, down regulation of TTR mRNA and protein upon differentiation was associated with significant up regulation of both miR200a-3p and miR-141-3p (Fig. 3f). However, the fold change of miR-141-3p was remarkably higher in differentiated cells compared to the fold change of miR-200a-3p.

### Ectopic overexpression of regulatory microRNAs curtails endogenous TTR levels and Thyroxin uptake in human trophoblast cells

**Figure 4:**
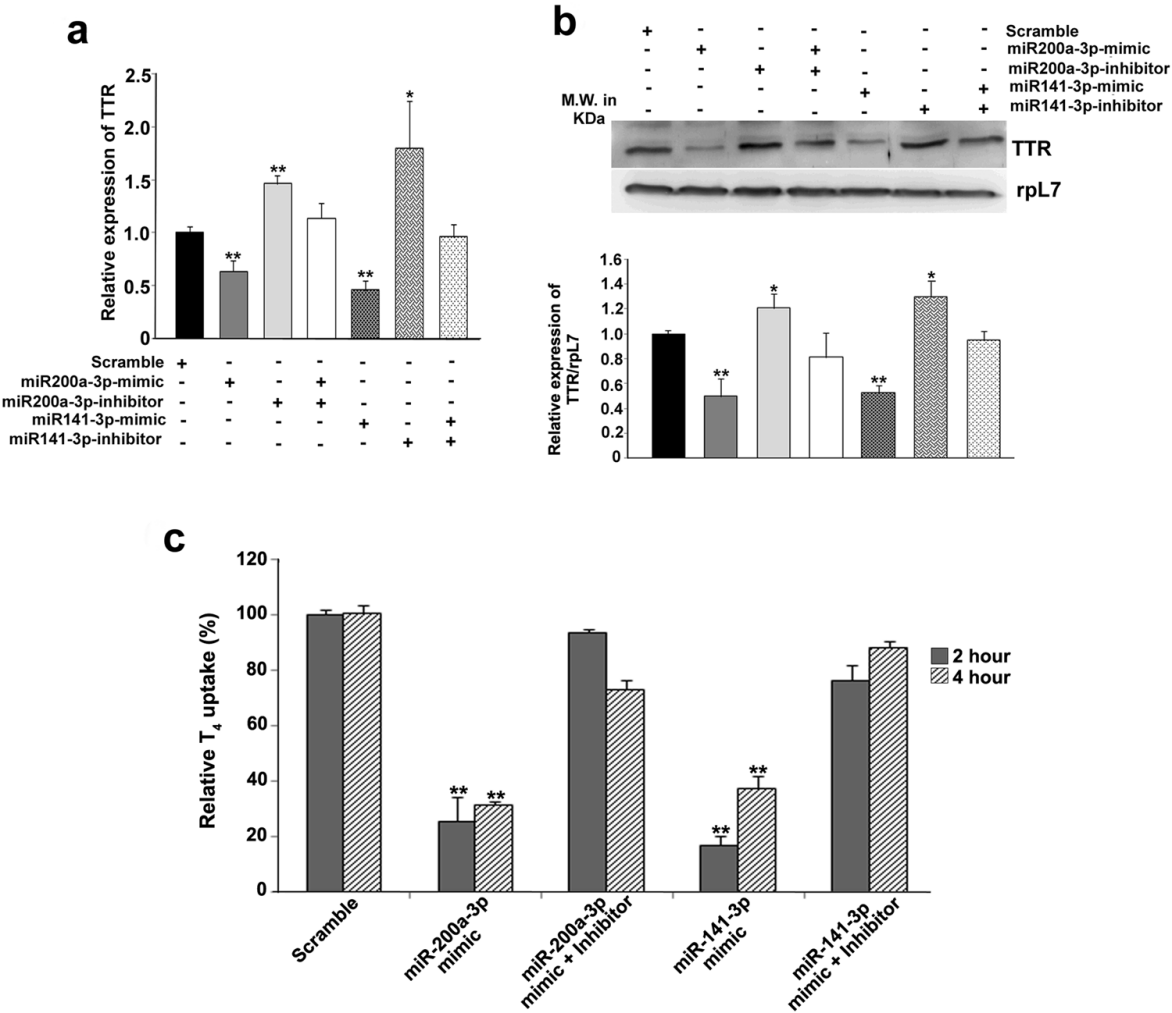
Overexpression and inhibition of miR-200a-3p and miR-141-3p regulate TTR and its function in human trophoblast cells. (**a**) Quantitative real time PCR of TTR in BeWo cells transfected with either mimic/inhibitor alone or in combination of mimic with the respective inhibitor for miR-141-3p and miR-200a-3p. (**b**) Western blot analysis of TTR protein from BeWo cells transfected with either mimic/inhibitor alone or in combination of mimic with the respective inhibitor for miR-141-3p and miR-200a-3p. Eighty microgram of total cellular protein was loaded in each lane. RpL7 was used as loading control. Densitometric quantification TTR protein level relative to rpL7 is shown below. Data presented are means and standard error of the mean of four replicates. (**c**) T_4_ uptake assay in BeWo cells, transfected with either microRNA mimic alone or mimic along with inhibitors of respective microRNAs. Relative T_4_ uptake was monitored by Sandell-Kolthoff reaction and results were normalized to the uptake value taken at 1 min interval period. Relative T_4_ uptake from BeWo cells transfected with scrambled mimic was taken as control. Results shown are mean ± SEM from four biological replicates. *p < 0.05, **p < 0.005.

To test whether gain in regulatory microRNAs in undifferentiated BeWo cells, where the expression of the microRNAs is relatively low, lead to change in TTR expression, mature miR-200a-3p or miR-141-3p mimics were transfected into BeWo cells in the absence or presence of the respective inhibitors. Scramble microRNA transfected cells were used as control. Over expression of either miR-200a-3p or miR-141-3p using mimics in undifferentiated BeWo cells led to reduced expression of TTR transcript. These silencing effects of microRNA mimics were found to be reversed by the addition of respective microRNA inhibitors (Fig. 4a). Similar results were obtained for TTR protein levels (Fig. 4b). This data clearly indicates that both miR-200a-3p and miR-141-3p have the ability to regulate endogenous TTR protein levels in human trophoblast cells.

TTR, produced by placental trophoblast cells, plays crucial role in transplacental delivery of thyroxin hormone (T_4_) from maternal blood to the growing fetus. Our data revealed that overexpression of miR-200a-3p or miR-141-3p repressed the expression of TTR in human trophoblast cells. To investigate the functional consequences of microRNA-mediated silencing of this T_4_-carrier protein, the role of these two microRNAs on uptake of T_4_ hormone was assessed in BeWo cells. Overexpression of either miR200a-3p or 141-3p led to almost 80% decrease in T_4_ uptake by BeWo cells as early as 2 h, which persisted through 4 h time point as compared to scramble transfected cells (Fig. 4c). As expected, addition of the corresponding microRNA inhibitor reversed the effect regaining the T_4_ uptake ability of BeWo cells at both time points (Fig. 4c). These data strongly suggest that miR-200a-3p and miR-141-3p may regulate the expression of TTR and consequently TTR-mediated delivery of thyroxin hormone in placental trophoblast cells.

### Intra-uterine growth restriction is associated with decreased TTR expression and concomitant increase in the regulatory microRNAs in rat placenta

**Figure 5:**
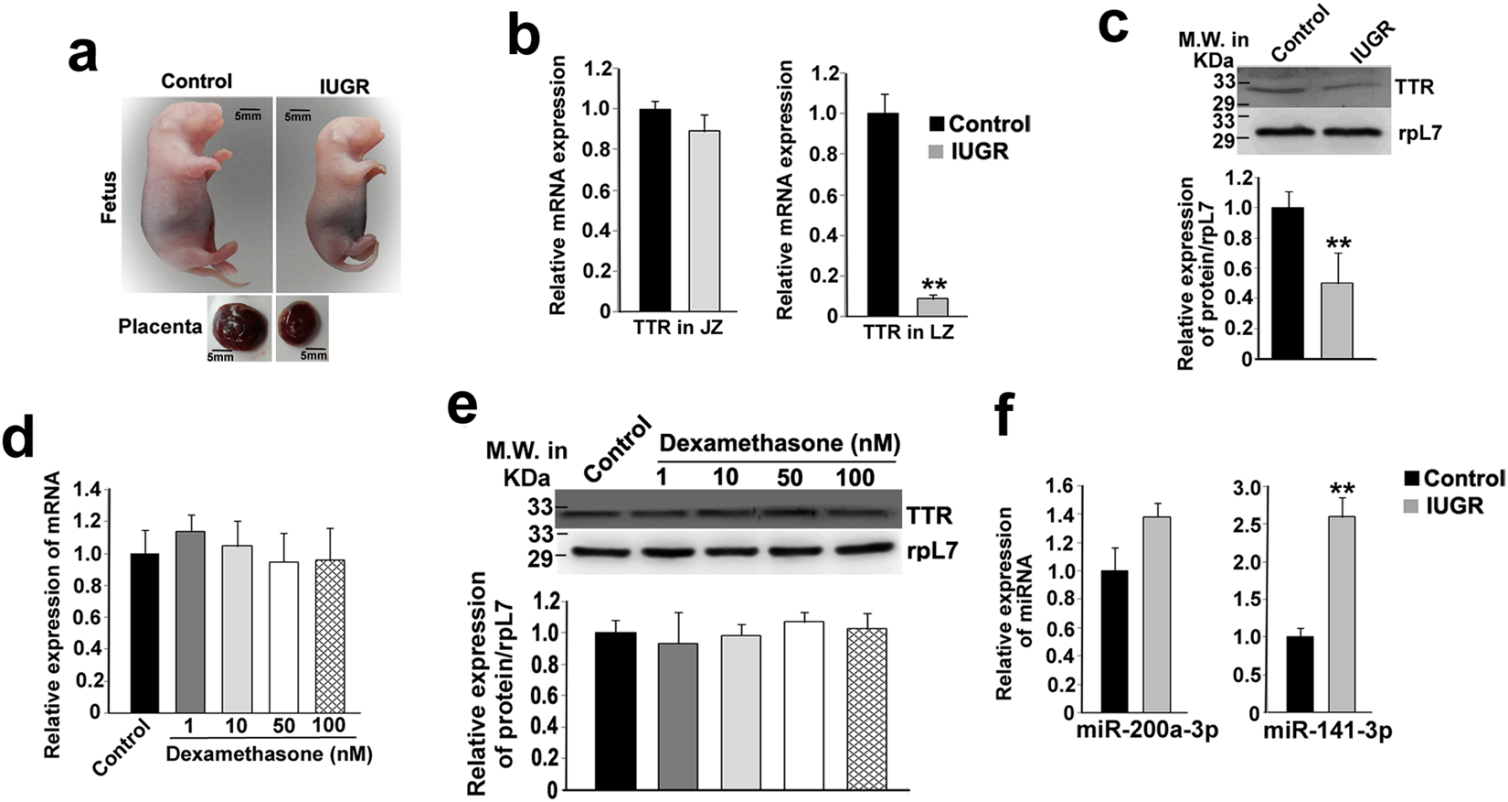
Induction of IUGR during pregnancy in rats impacts TTR and the regulatory microRNAs, miR-200a-3p and miR-141-3p. (**a**) Photographic images of control and dexamethasone-induced IUGR rat fetuses and placentas. (**b**) Quantitative real time PCR analysis of TTR mRNA from junctional and labyrinth zone of the control and IUGR rat placenta. rpL7 was used as endogenous control. (**c**) Western blot analysis of TTR protein in labyrinth zone from control and IUGR-induced rat placenta. Quantification of TTR protein by ImageJ software relative to RpL7 (Panel below). (**d**) Quantitative real time PCR analysis analysis of human TTR transcripts (hTTR) in BeWo cells treated with different doses of dexamethasone (1 nM–100 nM). (**e**) Western blot analysis of hTTR in vehicle (control) and dexamethasone treated BeWo. Densitometric analysis of hTTR protein in the representative western blot (Panel below). Asterisks (**) indicate significant differences (p < 0.005) compared to control. (**f**) Taqman assays for miR-200a-3p and miR-141-3p in labyrinth zone from control and IUGR-induced rat placenta. rpL7 and U6 snRNA was used as endogenous control. Experiments were performed using placenta from five different groups of control and IUGR rats (n = 5).

Restriction in placental growth has direct impact on the development of the growing fetus. Since compromised thyroxin uptake might affect fetal metabolism during pregnancy and consequently lead to IUGR, the expression of TTR was analyzed in the placenta from an IUGR rat model. IUGR was induced in rats by dexamethasone administration during days 13.5 to 20.5 of gestation as reported previously. As expected, dexamethasone treatment resulted in retardation of both fetal and placental growth without affecting fetal viability and number of fetuses (Fig. 5a, Table 1).

**Table 1:** Maternal dexamethasone administration induces IUGR and impacts fetal and placental development in rats.

	Control group (vehicle treated)^a^ (n = 5)	Dexamethasone-treated group^a^ (n = 5)	P-value
Number of pups	12 ± 1.73	13 ± 1.53	ns
Fetal weight (gm)	3.85 ± 0.5	2.47 ± 0.4	*p* < 0.0001
Placental weight (gm)	0.414 ± 0.08	0.25 ± 0.06	*p* < 0.001

^a^Values indicate mean ± SEM; ns: non-significant (p > 0.05).

The influence of IUGR on the expression of TTR mRNA was dependent on the placental zone. Induction of IUGR during pregnancy had no effect on TTR mRNA levels in the junctional zone of the placenta (Fig. 5b; Ct values ~26.5 in both control and IUGR placentae). In contrast, there was massive down regulation of TTR transcripts in the labyrinth zone of the IUGR placentae as compared to controls (Fig. 5b, ~90%). Decrease in TTR levels in the labyrinth zone of the placenta upon IUGR induction was further confirmed by western blot followed by densitometric analysis (Fig. 5c). In order to address whether the effect on TTR expression in placenta is due to exposure of dexamethasone or it is a real consequence of IUGR, BeWo trophoblast cells were treated with various doses of dexamethasone and TTR expression was analyzed. Both mRNA and protein levels of TTR was unaffected in trophoblast cells by dexamethasone treatment (Fig. 5d and e). In summary, dexamethasone-induced IUGR affected TTR expression in the fetal placenta. This result prompted us to analyze whether the regulatory microRNAs that were found to bind and regulate TTR expression and thyroxin uptake in trophoblast cells, are also affected by IUGR induction. As expected, miR-141-3p was significantly up regulated in the labyrinth zone of the IUGR placentae as compared to control. On the contrary, miR-200a-3p did not show any significant change between control and IUGR placentae (Fig. 5f).

### MiR-141-3p is highly expressed with concurrent down regulation of TTR in human IUGR fetal placenta

**Figure 6:**
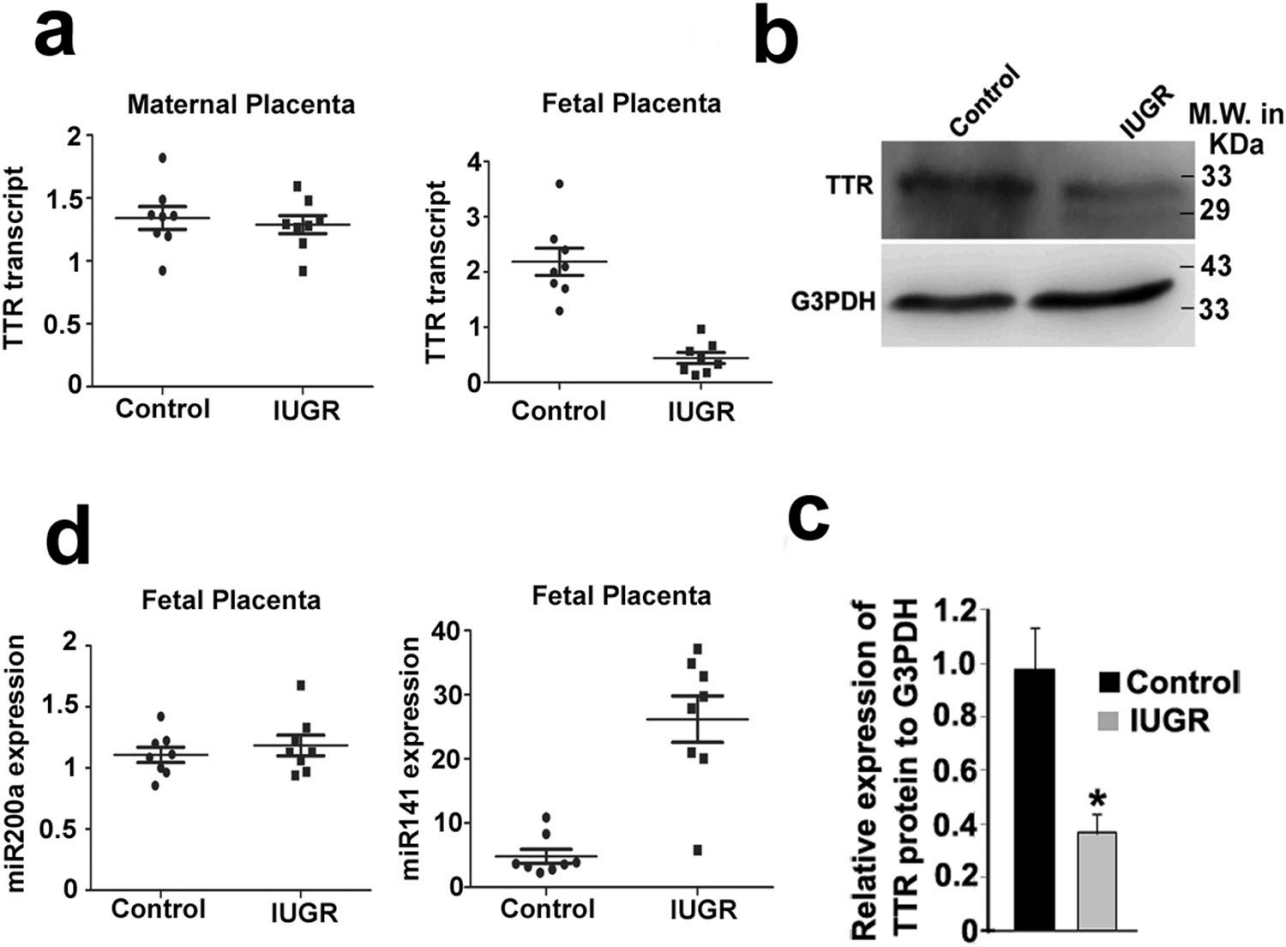
MiR-141-3p and TTR are reciprocally expressed in the human fetal placenta from IUGR patients. (**a**) Quantitative real time PCR analysis of TTR transcript using RNA from maternal and fetal placental samples collected from control and IUGR patients. (**b**) Western blot analysis of TTR in fetal placenta from control and IUGR patients. G3PDH was used as loading control. (**c**) Quantification by ImageJ software of the TTR protein relative to RpL7 using three biological replicates from b. (**d**) TaqMan assays for miR-200a-3p and miR-141-3p using RNA from the control and IUGR fetal placenta. U6 snRNA was used as endogenous control. Experiments were conducted using placenta from 8 different groups (n = 8). *p < 0.05.

To further establish the impact of IUGR on TTR and the regulatory microRNAs, we sought to analyze the TTR transcript and protein levels in term placenta from women with normal and IUGR pregnancy. Term placental samples were collected from maternal age matched 8 IUGR and 8 normal pregnancies. Clinical details of patients are listed in Table 2. Real time PCR analysis showed that there was negligible expression of TTR in the maternal placentae (Ct ≥ 30 in all samples analyzed). However, in the fetal placenta, both TTR mRNA and protein were found to be substantially expressed in control placentae (Fig. 6a,b and c) by both real time PCR and western blot analysis. However, in IUGR placentae, there was significant decrease in TTR expression as compared to control (Fig. 6a,b and c). Interestingly, the level of miR-200a-3p was negligible in the both control and IUGR fetal placentae, whereas, miR-141-3p levels were high in the IUGR fetal placentae as compared to controls (6d).

**Table 2:** Clinical characteristics of human subject.

	Control (n = 8)	IUGR (n = 8)	P-value
Maternal age (y)	24.2 ± 2.68	21.25 ± 1.25	ns
Gestational age at delivery (wks)	37.78 ± 0.48	33.63 ± 1.26	p < 0.01
Gravidity	1.5 ± 0.76	1.62 ± 0.74	ns
Birth weight (g)	2718 ± 175	1581 ± 231	p < 0.01

Values expressed as mean ± SEM. ns: non-significant (p > 0.05).

## Discussion

Transthyretin (TTR) has been reported to be vital for normal embryonic growth during pregnancy^45^, yet the molecular regulation of TTR biogenesis has remained elusive. The sites and cellular origin of TTR synthesis are rather diverse^46,47^, but in line with its role in proper embryo development, it is abundantly expressed by placental villous trophoblast cells in human^13,14^. Although the expression of TTR in IUGR and preeclampsia is controversial^20–25^, arguments are in favour of low TTR levels in IUGR/preeclampsia. MicroRNAs have been shown to play significant roles in regulating genes important for fetal development during pregnancy^35–37,48^. Besides, manipulation of TTR levels under pathophysiological conditions using siRNA approach prompted the current investigation into the roles of microRNAs in regulating TTR biogenesis in trophoblast cells during normal and IUGR pregnancies.

Our data showed that TTR expression is high in the last third of gestation in rats and remained so till the end of gestation similar to TTR expression reported previously in human^15^. Interestingly, our data confirmed that TTR expression is not restricted to any particular lineage of trophoblast cells indicating that high rate of metabolism needed in placental trophoblast cells is driven by higher abundance of thyroxin-mediated transcription in placental trophoblast cells^49^. Our study on conserved microRNAs as plausible regulators of TTR biogenesis led to identification of two microRNAs, miR-200a-3p and miR-141-3p that directly bind to the 3′-UTR of TTR. Furthermore, our results on the abrogation of binding of these microRNAs to the 3′-UTR of TTR by mutating the microRNA response elements indicates specificity of these microRNA binding to the 3′-UTR of TTR. Transcriptional regulation of TTR in various cell lines by estrogen receptor-α and -β, hepatocyte nuclear factor-3 and 4α is well documented in literature^50–53^. Our finding that miR-141-3p and miR-200a-3p are involved in the regulation of TTR during trophoblast differentiation provides yet another mechanism by which microRNA adds a complementary layer of control at the post-transcriptional level to developmentally indispensible genes known to be transcriptionally regulated.

Our further efforts were focused on elucidating the role of these regulatory microRNAs during trophoblast differentiation. Since presence of microRNA binding sites and microRNAs being able to specifically bind to 3′-UTR of a transcript not always endorse the role of such microRNAs in regulation of the gene in physiological context, we attempted to illustrate the functional significance of these regulatory microRNAs in human trophoblast differentiation. In human, fusion of trophoblast cells is the key event of placental development and maturation. During human placental development, mononucleated cytotrophoblast cells undergo syncytialization to form multinucleated syncytiotrophoblast which ultimately form the outermost layer of the placental villi, where it comes into direct contact of maternal blood. In later stage of pregnancy this syncytiotrophoblast plays important roles in hormone production, immunotolerence and nutrient exchange^54–56^. In order to test the role of the regulatory microRNAs in differentiation (Syncytitialization), differentiation was induced in BeWo cells using 8-Bromo-cAMP. Syncytialization of BeWo results in down regulation of TTR transcript as well protein, implying that cytotrophoblast pool is the main source of TTR secretion in human placenta. Significant up-regulation of both miR-141-3p and miR-200a-3p levels was observed along with TTR down regulation in differentiating human trophoblast cells. These data indicate that miR141-3p and miR-200a-3p might play important roles in regulating TTR during normal placental development. Interestingly, it has recently been demonstrated that miR-141-3p affects proliferation and invasion of two different trophoblastic cell lines, JEG-3 and HTR-8/SVneo, which are considered as first and third trimester trophoblast cells, respectively^42^. However, we could not detect any significant expression of TTR transcript in JEG-3 and HTR-8/SVneo cells (Ct > 35). These results along with our data indicate that miR-141-3p might function differently in different cellular and physiological context.

Our data on overexpression of the microRNA mimics and their respective inhibitors in BeWo cells reveal that these microRNAs not only down regulate TTR specifically but also curtail its function of T_4_ uptake by trophoblast cells. Since TTR biogenesis by placental trophoblast cells is crucial for proper fetal development, our results on miR-141-3p and miR-200a-3p in regulating T_4_ uptake by human trophoblast cells led to next set of experiments using IUGR rat model as well as in human IUGR placentas. Our data revealed that TTR transcripts are massively down regulated in the labyrinth zone of rat IUGR placenta, whereas, it remained unaltered in the junctional zone of the placenta. Our data therefore, reinforce the importance of labyrinth-derived TTR in trans-placental passage of T_4_ in addition to boosting overall metabolism in the placental trophoblast. In agreement with these data, miR-141-3p was found to be up-regulated in labyrinth zone of IUGR rat placenta. In contrast, miR-200a-3p remained unchanged in labyrinth zone of the placenta in control and IUGR rats indicating that miR-200a-3p is not physiologically relevant during IUGR in rat. Leading on from this, we tested the role of miR-141-3p and miR-200a-3p in term placenta from human control and IUGR pregnancies. Interestingly, our data highlight that in human IUGR placenta the regulation of TTR by miR-141-3p and not miR-200a-3p is physiologically relevant like that in rat.

Recent studies reported that miR-141-3p, belonging to the miR-200 cluster, is overexpressed in IUGR as well as in preeclamptic placentas^42–44^. They also have shown that in IUGR patients, elevated concentration of miR-141-3p can repress PLAG1 at both transcriptional and posttranscriptional levels. It was further demonstrated by our group that miR-141-3p regulates IGF2 during mouse placental development^48^. These data are rather intriguing since miR-141-3p emerges as a central regulator of various genes pivotal for proper embryonic development and can be used as a potential biomarker as well as therapeutic target to combat IUGR.

In summary, we identified TTR as a direct target of miR-141-3p or miR-200a-3p and demonstrated that TTR and these microRNAs are reciprocally expressed during human trophoblast differentiation. MiR-141-3p or miR-200a-3p can regulate endogenous TTR protein levels as well as thyroxin uptake by human trophoblast cells. On the contrary, under pathophysiological conditions, such as, IUGR, only miR-141-3p is relevant and it regulates TTR levels both in rats and human during IUGR. Understanding of how microRNAs control biogenesis of TTR, a key molecule in regulating fetal growth, will help us to uncover the underlying causes of placental insufficiency and IUGR.

## Methods

### Animals and tissue preparation

To obtain rat placental tissues, sexually matured female Sprague-Dawley rats obtained from Indian Institute of Chemical Biology (IICB) animal house were caged overnight with fertile males. Day 0.5 of pregnancy was designated by the presence of sperm in morning vaginal smears. Imaplantation sites were dissected out and frozen in dry ice-cooled hexane for *in situ* hybridization. Intra-uterine growth restriction (IUGR) was induced in rats as described previously^57–59^. On day 13.5 of pregnancy, pregnant rats were subcutaneously injected with a bolus dose of 100 μg dexamethasone acetate (Sigma, St Louis, MO, USA) in 0.1 ml 10% ethanol or with the vehicle only and used as controls. Animals were then anesthetized with ketamine/xylazine, and an alzet osmotic pump (Durect Corp., USA) was subcutaneously implanted. The osmotic pumps were calibrated to release 200 μg dexamethasone acetate/kg maternal body weight/day. Control rats received osmotic pumps containing vehicle. Five animals were used in each group. Animals were sacrificed on day 20.5 of pregnancy. Placental tissues (junctional zone and labyrinth zone) were dissected from pregnant animals. Tissues were snap-frozen in liquid nitrogen for RNA and protein analysis. For *in situ* hybridization, tissues were frozen in dry ice-cooled hexane. All tissue samples were stored at −80 °C until used. The Indian Institute of Chemical Biology Animal Care and Use Committee (registration number 147/1999/CPCSEA) approved all procedures for handling and experimentation with rats. The use of rats was conducted in accordance with the regulations set forward by the Committee for the Purpose of Control and Supervision of Experiments on Animals, Govt. of India (http://cpcsea.nic.in). All the techniques/procedures were designed to provide for maximum comfort/minimal stress to the animals.

### Human sample collection

Human term placental tissue samples were collected from mothers with normal sized baby (control) and mothers with Intra-Uterine Growth Retarded (IUGR) baby. These samples were obtained from Calcutta National Medical College & Hospital (CNMC), post delivery. The placentas were placed on sterile container and processed within 4 h following delivery. The outer sac was removed carefully. From the maternal side of the cotyledons, 0.5 cm tissue was dissected out carefully and was termed as maternal placenta as per standard protocol. Fetal placenta was collected from the same cotyledon. Small pieces of tissue were cut both from the fetal and maternal placenta, rinsed in 0.9% sterile PBS to remove attached blood and other impurities. Tissues were snap-frozen in liquid nitrogen for RNA and protein isolation and kept at −80 °C until used. Written informed consent was obtained from all subject and documented. All experimental protocols that used human tissue were approved by Institutional Ethics Committee for Human Research, Calcutta National Medical College, India in accordance with the relevant guidelines and regulations set forward by Indian Council of Medical Research (http://icmr.nic.in/human_ethics.htm).

### Cell culture, transfection and differentiation

Human trophoblast cell line, BeWo and HEK293 cell line were purchased from American Type Culture Collection (USA). BeWo cells were maintained in F-12K Ham (Sigma, USA), supplemented with 10% fetal bovine serum (Invitrogen, USA) and were cultured in a humidified atmosphere of 5% CO_2_. For dexamethasone treatment, BeWo cells were grown to 60% confluency, and then were cultured in complete growth medium supplemented with dexamethasone at various concentrations ranging from 1–100 nM. Control cells were treated with similar dose of vehicle (ethanol). After 48 hours of treatment the cells were harvested and used for RNA and protein isolation.

For transfection experiments, BeWo cells were seeded on 35 mm culture dishes (BD-Falcon, USA) 24 hours prior to transfection. Cells were transfected with microRNA mimics for miR-141-3p or miR-200a-3p (Ambion, USA) individually at a final concentration of 200 nM (titrated for maximum down regulation of TTR prior to this experiment, data not shown) using Lipofectamine RNAiMAX (Invitrogen, USA) as per manufacturer’s instructions. Similarly microRNA inhibitors for miR-141-3p or miR-200a-3p (Ambion, USA) were transfected at a final concentration of 200 nM to prove the specificity of the microRNA action. Both mimic and inhibitor were also used for transfection in a separate treatment. RNA and protein were isolated after 48 hours of transfection. For negative control samples cells were transfected with scramble mimics.

For induction of differentiation (syncytialization) in BeWo, cells were allowed to grow normally in culture dishes and on cover slips upto 40% confluency. Then the cells were treated with 8-Bromo-cAMP (Sigma, USA) at a final concentration of 250 µM for 72 hours. Cells were then harvested for isolation of RNA or protein and cells grown on coverslips, were used for phalloidin-hoechst staining.

### ***In situ*** hybridization

*In situ* hybridization was performed using digoxigenin-labeled cRNA probes as described previously^48,60^.

### Phalloidin-Hoechst staining of cells

To visualize the formation of syncytium, BeWo cells were grown on coverslip in a 35 mm dish in the absence and presence of 8-Bromo-cAMP and fixed with freshly prepared ice-cold 4% para-formaldehyde for 15 minutes at room temperature followed by three PBS washes (5 minutes each). To stain the cytoskeleton, fixed cells were incubated with phalloidin (Cell Signaling Technology) at 1:200 dilution for 15 minutes. Then the cells were again washed with PBS for three times and incubated with Hoechst at a final concentration of 2 mg/ml for another 15 minutes to stain the nuclei. Then the cells were rinsed with PBS for several times and finally, the coverslip was mounted on a glass slide with the help of fluoroshield (Sigma, USA) and examined under Leica epifluorescence microscope.

### RNA preparation and quantitative real time PCR

Total RNA was extracted from either tissue or BeWo cells by using TRIzol reagent (Invitrogen, USA), as per the manufacturer’s protocol. 5 µg of total RNA was reverse transcribed using the M-MLV Reverse Transcription kit (Invitrogen, USA). Ten-fold dilution of cDNAs and FG-Power SYBR green PCR Master Mix (Applied Biosystems, USA) was used in the real time PCR reaction using 7500 Real-Time PCR System (Applied Biosystems, USA). PCR Conditions included initial holding stage (95 °C for 10 min) and 40 cycles (95 °C for 15 s and 60 °C for 1 min) followed by a dissociation stage (95 °C for 15 s, 60 °C for 1 min, and then 95 °C for 30 s). Primers specific for genes of interest are listed in Table 3. Expression of rpl7 RNA was used as an internal control. At least three different biological replicates were used for every reaction. Samples were normalized to the control sample for each gene.

**Table 3:** Primers used for real time-PCR analysis.

No.	Gene name	Forward primer (5′ to 3′)	Reverse primer (5′ to 3′)
1	hTTR	GATGACACCTGGGAGCCATT	ATGCCAAGTGCCTTCCAGTAA
2	rTTR	CACGGGCTCACCACAGATG	GTAGTGGCGATGACCAGAGTCA
3	hCG-β	GTCAACACCACCATCTGTGC	GGCCTTTGAGGAAGAGGAGT
4	hSynsytin2	CCAGCTACCTGGGCATATCAG	GGAGGCATTGGTGAATCGA
5	hRPL-7	AATGGCGAGGATGGCAAGA	AAGGCGAAGAAGCTGCAACA
6	rRpl-7	CCAGCTACCTGGGCATATCAG	GGAGGCATTGGTGAATCGAC

For quantitative analysis of microRNAs, total RNA was isolated using miRVana RNA isolation kit (Ambion, USA). 50ng of total RNA was reverse transcribed using specific RT primers for miR-141-3p, miR-200a-3p or U6 snRNA (U6, Assay ID 001973; miR-141-3p, Assay ID 000463; miR-200a-3p, Assay ID 000502), using a TaqMan Micro-RNA RT Kit (Applied Biosystems, USA). Expression of each microRNA was determined by TaqMan assay using specific TaqMan probes and TaqMan Universal PCR Master Mix (Applied Biosystems, USA) with a standard thermal cycling condition which includes initial denaturation at 95 °C for 10 min, followed by 40 cycles of denaturation for 15 s at 95 °C, annealing and extension for 1 min at 60 °C. The microRNA levels were normalized to U6 snRNA expression level. Samples were analyzed in triplicates from minimum three biological replicates. The amount of both RNA and microRNA was normalized relative to the amount of endogenous control (∆∆Ct = ∆Ct_RNA_ − ∆Ct _Rpl7_ for RNA and ∆∆Ct = ∆Ct_microRNA_ − ∆Ct_U6_ for microRNAs).

For patient samples, TTR and microRNA expression were quantified by real time PCR as described above using a 96-well-array format. Each expression data was normalized on endogenous levels of Rpl7 (for TTR) or U6 snRNA (for microRNAs) and expression fold change were determined by 2^−∆∆Ct^ method as described above. Each sample was analysed in triplicate. Data were represented by column scatter plot using GraphPad Prism v. 5.04.

### Reporter plasmid construction

Human TTR (hTTR) 3′UTR 250-nt fragment (14–263 bp) containing the binding site for miR-141-3p and miR-200a-3p (5′-CAGTGTT-3′) was amplified from genomic DNA by high-fidelity LA-Taq kit (Takara) using the following primer pair: forward primer: 5′-ATATGCTAGCCAGTGGACCTGAAGGACGAG-3′ and reverse primer: 5′- ATTAGTCGACGCGTTCTGCCCAGATACTTT-3′. The mutated version of hTTR 3′UTR was generated by using Phusion Site-Directed Mutagenesis Kit (Thermo Scientific, USA) according to the manufacturer’s instructions. This mutagenic reaction introduced two point mutations in the binding site of 141-3p and miR-200a-3p (5′-CGGTGCT-3′). The primers used for the site directed mutagenesis in the binding site for the microRNAs were: forward primer: 5′-TTTACTAAAGCGG TGCTTTCACCTCAT-3′ and reverse primer: 5′-AGTAAAAATGGAATACTCTTGGTT ACA-3′. The amplified products were then cloned in pmirGLO vector (Promega, USA) downstream of the firefly luciferase gene using NheI and SalI sites to generate ‘wild type’ and ‘mutated’ luciferase reporter plasmids. Both of these reporter plasmids were used for luciferase assay.

### Dual luciferase assay

Dual luciferase assay was performed as described previously^48^. Briefly, HEK-293 cells were seeded at 7 × 10^4^ cells/ well in 96-well plate 24 hours prior to transfection. For control sample, 150 ng pmirGLO reporter plasmid (Promega, USA) containing either wild type or mutated 3′UTR of hTTR was transfected along with 75 nM scrambled mimic. For test samples, cells were transfected with 150ng reporter plasmid along with 75 nM microRNA-mimic alone or 75 nM microRNA-mimic plus 100 nM microRNA inhibitor. Lipofectamine LTX and Lipofectamine RNAiMAX (Life- Technologies, USA) were used for transfection of plasmid DNA and microRNA (mimics and/or inhibitors) respectively. Firefly and renilla luciferase signals were measured 24 hours following transfection using a multimode plate reader (Perkin Elmer, USA) and Dual-Luciferase Reporter Assay kit (Promega, USA) as per manufacturer’s protocol. Renilla luciferase activity was used for transfection normalization. Relative fold suppression of luciferase activity was determined by taking the activity of control sample as 100%. All experiments were performed in triplicates using at least three different biological replicates and the data were presented as the mean ± SEM.

### Western blot analysis

Western blotting was performed as described previously^61^. Total protein was extracted from tissue or cells using RIPA buffer. Protein concentration for each sample was estimated by using the Bio-Rad Protein Assay reagent (Bio-Rad, USA). 80–100 µg of total proteins were separated by 10–12% SDS-PAGE under reducing condition and were then transferred to PVDF membranes (Millipore, USA). The blots were then blocked for 1 hour in 5% skim milk in TBS-T. Rabbit anti-TTR (Santa Cruz Biotechnology) and Rabbit anti-RPL-7 (Bethyl Lab) antibodies were used at recommended dilutions. The blots were incubated in primary antibody solution for overnight at 4 °C. Then the blots were incubated in HRP-conjugated goat anti-rabbit IgG (Cell signaling Technology) diluted 1:2000 in TBS-T for 1.5 hours at room temperature. Blots were developed using an ECL kit (Millipore) according manufacture’s protocol and images were captured by Biospectrum 810 imaging system (UVP, USA). Densitometric analysis was done by NIH ImageJ software. Three to five biological replicates were used for each experiment.

### Non-radioactive T_4_ uptake assay

Uptake assay for thyroxin hormone (T4) was performed with microRNA mimic or mimic plus inhibitor transfected BeWo cells by a non-radioactive assay procedure as described in Jayarama-Naidu *et al*.^62^. In brief, BeWo cells were transfected with miR-141-3p or miR-200a-3p mimic alone or mimic plus inhibitors as described above. After 10 hours of transfection the cells were trypsinized and plated at 1 × 10^4^ cells/well density onto 96-well culture plate and allowed to grow for next 48 hours. The cells were then serum starved for another 12 hours following which uptake assay was performed. For uptake assay, cells were washed with PBS at room temperature followed by incubation with 10 µM T_4_ (Sigma, USA) in uptake buffer (HEPES, pH 7.4; supplemented with 125 mM NaCl, 1.3 mM CaCl_2_, 1.2 mM MgCl_2_, 5 mM KCl and 5.6 mM D-glucose) either for 1 minute or 2 hours or 4 hours. Control cells were treated with vehicle in uptake buffer. Following the incubation period the medium was discarded and the cells were washed twice with PBS-0.1% BSA. Cells were then incubated with 50 µl 0.68 M ammonium persulfate (APS) in water for 1 h at 90 °C. To 20 µl of this digest 50 μl of Ceric solution [25 mM (NH_4_)_4_Ce(SO_4_)_4_ and 0.5 M H_2_SO_4_] and 50 μl of Arsenous solution (25 mM NaAsO_2_, 0.5 M H_2_SO_4_ and 0.2 M NaCl) were then added. The iodide moiety generated by the APS oxidation acts as a catalyst in the reduction of ceric ammonium sulfate (yellow color) to the cerous form (colorless) in the presence of arsenious acid (Sandell-Kolthoff Reaction) and was quantified by measuring absorption at 415 nm. The relative T_4_ uptake % was calculated as [(OD_1min_ − OD_2h or 4h_)/OD_1min_] × 100.

### Bioinformatic analysis

The putative binding sites for miR-141-3p and miR-200a-3p on 3′-UTRs of human and rat TTR were identified using the TargetScan mouse 6.0, PicTar, microRNA.org, miRWalk, miRDB, MicroCosm Target and DIANA microT 3.0 search engines. Conserved microRNAs were selected for further validation if they were predicted by at least 5 databases mentioned above.

### Statistical analysis

Each experiment was repeated at least three times with different biological samples unless mentioned otherwise. The data were analyzed by ANOVA followed by Newman-Keuls multiple comparison test.
